# The First Complete Mitochondrial Genome of *Eucrate crenata* (Decapoda: Brachyura: Goneplacidae) and Phylogenetic Relationships within Infraorder Brachyura

**DOI:** 10.3390/genes13071127

**Published:** 2022-06-23

**Authors:** Xiaoke Pang, Chenglong Han, Biao Guo, Kefeng Liu, Xiaolong Lin, Xueqiang Lu

**Affiliations:** 1 Tianjin Key Laboratory of Environmental Technology for Complex Trans-Media Pollution and Tianjin International Joint Research Center for Environmental Biogeochemical Technology, College of Environmental Science and Engineering, Nankai University, Tianjin 300350, China; Iris13205415829@126.com (X.P.); hanchenglong185@163.com (C.H.); 2 Tianjin Fisheries Research Institute, Tianjin 300457, China; oucguobiao@163.com (B.G.); liukefeng@sina.com (K.L.); 3 Shanghai Universities Key Laboratory of Marine Animal Taxonomy and Evolution, Shanghai Ocean University, Shanghai 201306, China; 4 Engineering Research Center of Environmental DNA and Ecological Water Health Assessment, Shanghai Ocean University, Shanghai 201306, China

**Keywords:** mitochondrial genome, *Eucrate crenata*, Goneplacidae, Brachyura, phylogeny

## Abstract

Characterizing the complete mitochondrial genome (mitogenome) of an organism is useful for genomic studies in taxonomy and evolution. The mitogenomic characteristics of *Eucrate crenata* (Decapoda: Brachyura: Goneplacidae) have never been studied. The present study decodes the first mitogenome of *E. crenata* by high-throughput sequencing (HTS). The length of the mitogenome is 15,597 bp, and it contains 13 protein-coding genes, 2 ribosomal RNA genes (*rrnS* and *rrnL*), and 22 transfer RNA genes. There are 14 and 23 genes observed on the heavy and light strands, respectively. *E. crenata* possesses a *trnH-cac* translocation, with the *trnH-cac* shifted between *trnE-gaa* and *trnF-ttc* instead of the usual location between *nad5* and *nad4* in decapods. Phylogenetic analyses based on the current dataset of 33 Brachyuran mitogenomes indicate that *E. crenata*. is closely related to *Ashtoret lunaris* of Matutidae. The similar codon usage and rearrangements in the two species provide evidence for their close phylogenetic relationship. Positive selection analysis showed that one residue located in *cox1* was identified as a positively selected site with high BEB value (>95%), indicating that this gene was under positive selection pressure. This study is the first complete mitogenome record for the family Goneplacidae, and the results obtained may improve the understanding of the phylogeny of Goneplacidae in Brachyura.

## 1. Introduction

Mitochondria are involved in various processes, such as ATP generation, signaling, cell differentiation, growth, and apoptosis [1]. Based on this, the mitochondrial genome (mitogenome) has been widely used in studies of molecular evolution and the reconstruction of phylogeny [2,3]. Nucleotide sequences or amino acid data, and the gene arrangement of mitogenomes, are greatly useful for inferring relationships among metazoan lineages [3,4]. In general, a metazoan mitogenome is a closed circular DNA molecule, ranging in size from 14 to 20 kb, and typically contains 37 genes: 13 protein-coding genes (PCGs) (*atp6*, *atp8*, *cox1-3*, *cob*, *nad1-6*, and *nad4l*), 2 rRNA genes (*rrnS* and *rrnL*), 22 tRNA genes, and an AT-rich region (also known as the control region) that contains some of the initiation sites for genome transcription and replication [5]. The structural characteristics and arrangements of these genomic features of mitogenome differ across each species [6,7]. Mitogenomic composition can thus be examined for species identification, genetic diversity assessment, phylogenetics, and selection pressure analysis at various taxonomic levels [8,9]. Therefore, this may be helpful in revealing evidence of energy metabolism regulation mechanisms of species adapting to a specific environment.

The Brachyura crab is the largest clade of the Decapoda crustacean group, with more than 7250 known species, including 98 marine, freshwater, and terrestrial habitat families [10]. Marine Brachyura has a wide range of morphological and ecological diversity, leading to a complex taxonomy [11]. Goneplacidae belongs to the infraorder Brachyura and is known for its morphological complexity, and most are economically important. According to the literature [12], 66 species of the family Goneplacidae have been reported in total. Furthermore, based on ecological characteristics, economic values, and market values, these species in Goneplacidae are not only key functional species for strengthening the fishery community structure in aquatic ecosystem, but also important economic resources in marine pastures [13,14]. Thus, the investigations about the species mitogenomes in Goneplacidae and their phylogenetic relationships within the infraorder Brachyura are worthy of great attention. Up to now, the phylogenetic relationships within the infraorder Brachyura of several crabs belonging to some families, i.e., Grapsidae, Sesarmidae, Panopeidae, have been studied [15,16,17]. However, the species mitogenomes in the family Goneplacidae have never been reported, nor have their phylogenies within the infraorder Brachyura. *Eucrate crenata* (De Haan, 1835) (Decapoda: Brachyura: Goneplacidae) generally inhabits the sandy seabed with a depth of 10–100 m and occurs in the Korean Strait, the Red Sea, Japan, Thailand, India, and China. As an important bioindicator in Goneplacidae, most studies of this species focus solely on morphology and growth [18]. As reported, the carapace of *E. crenata* is cream-colored, with purple spots in the central area and distinctly pale anterolateral edges; and the outline is quadrilateral, slightly wider than the length [18].

Prior to this study, the mitogenomic characteristics of *E. crenata* have never been studied. We sequenced and annotated the first complete mitogenome of *E. crenata* based on specimen from the Bohai Bay in China. The mitogenome organization, codon usage, and gene order information of *E. crenata* were revealed. Combined with published data, the phylogenetic relationships of Brachyura were reconstructed based on mitogenomes to explore the potential status of Goneplacidae. We also performed positive selection analysis of Brachyura mitochondrial PCGs to understand the adaptive evolution of Brachyura.

## 2. Materials and Methods

### 2.1. Sample Collection, Identification and DNA Extraction

An adult of *E. crenata* (sample ID of LX2) was collected from Bohai Bay(38°32.12′ N, 118°1.50′ E), China in November 2021. Species-level morphological identification was carried out according to the main ideas of Nayak et al. [18]. The voucher specimen was preserved in 95% ethanol and stored at −80 °C until DNA extraction in the College of Environmental Science and Engineering at Nankai University (Tianjin, China). Genomic DNA was extracted from the first pinched muscle using the SDS method [19]. The harvested DNA was detected using agarose gel electrophoresis and quantified by a Qubit^®^ 2.0 Fluorometer (Thermo Scientific, Shanghai, China).

### 2.2. Illumina Sequencing, Mitogenome Assembly and Annotation

The DNA was first isolated, and then the 2 μg purified DNA was fragmented. Paired-end libraries (350 bp) were performed using the NEBNext^®^ Ultra™ DNA Library Prep Kit for Illumina (NEB, USA) following manufacturer’s instructions, then sequenced using an Illumina NovaSeq 6000 at the Beijing Novogene Bioinformatics Technology Co., Ltd. (Beijing, China).

Raw reads were filtered by Trimmomatic v0.39 before the assembly [20]. This filtering step was performed to remove the reads with connectors, the reads showing a quality score below 20 (Q < 20), the reads containing a percentage of uncalled bases (“N” characters) equal to or greater than 10%, and repeated sequences. A combination of de novo and reference-guided assemblies was applied to reconstruct the mitochondrial genome, and the following three steps were performed to assemble the mitogenome. First of all, the filtered reads were assembled into contigs with SPAdes 3.14.1 (http://bioinf.spbau.ru/spades, accessed on 30 December 2021). Then, BLAST (https://blast.ncbi.nlm.nih.gov/Blast.cgi, accessed on 30 December 2021) were used to align contigs with reference mitogenomes from the infraorder Brachyura species, and aligned contigs (≥80% similarity and query coverage) were ranked according to the reference mitogenomes. Finally, GapCloser 1.12 with default parameters were used to map the clean reads to the assembled draft mitogenome, and most gaps were filled by local assembly.

The mitochondrial genes were annotated using the online MITOS tool (http://mitos.bioinf.uni-leipzig.de/index.py, accessed on 30 December 2021), and the protein-coding genes, transfer RNA (tRNA) genes, and ribosome RNA (rRNA) genes were predicted with default parameters. BLAST searches for reference mitochondrion genes were used to determine the location of each coding gene. Manual corrections of genes for start/stop codons were carried out in SnapGene Viewer by referring to the reference mitogenomes. The circular map of mitogenome of *E. crenata* was drawn by the CGview tool (http://stothard.afns.ualberta.ca/cgview_server/, accessed on 30 December 2021). Several publicly available protein databases were functionally annotated using sequence-similarity Blast searches with a typical cut-off E-value of 10^−5^ against: NCBI non-redundant (Nr) protein database, Swiss-Prot, Clusters of Orthologous Groups (COGs), Kyoto Encyclopedia of Genes and Genomes (KEGG), and Gene Ontology (GO) terms.

### 2.3. Sequence Analysis

Relative synonymous codon usage (RSCU) was computed with cusp (EMBOSS v6.6.0.0). The AT and GC skews were calculated using the following formulas: AT skew = (A − T)/(A + T) and GC skew = (G − C)/(G + C) [21], where A, T, G and C refer to the occurrences of the four nucleotides. The complete mitochondrial DNA sequence has been deposited in GenBank with the accession number ON150678.

### 2.4. Phylogenetic Analysis

The phylogeny of the infraorder Brachyura was reconstructed using mitogenome data from 30 species (Table 1). Two species in the family Alpheidae were used as the outgroup (Table 1). Our data set was based on nucleotide sequences from 13 mitochondrial PCGs (*atp6*, *atp8*, *cox1*, *cox2*, *cox3*, *cob*, *nad1*, *nad2*, *nad3*, *nad4*, *nad4l*, *nad5*, and *nad6*). Multiple alignments of all 13 genes were performed with MUSCLE 3.8.31. In aligned sequences, blurred aligned regions and gaps were removed using Gblocks server 0.91b with the default setting. JModeltest v2.1.10 was used to select the most suitable evolutionary model GTR + I + G for the amino acid dataset. Maximum likelihood (ML) analysis of phylogenetic trees and model parameters was performed, and the best model was determined through AIC and BIC scores. The phylogenetic tree (http://www.atgc-montpellier.fr/phyml/, accessed on 23 February 2022) was constructed by PhyML v3.0 and graphically edited using the iTOL 3.4.3 (https://itol.embl.de/itol.cgi, accessed on 23 February 2022).

### 2.5. Positive Selection Analysis

The comparison of the nonsynonymous/synonymous substitution ratios (ω = dN/dS) is meaning for quantifying the impact of natural selection on molecular evolution [22]. Ω > 1, =1, and <1 respectively represent positive selection, neutrality, and purifying selection [23]. The codon-based maximum likelihood (CodeML) method, which implemented in the PAML package, [24] was used to estimate the ω values. A combined database of all 13 mitochondrial PCGs was used for selection pressure analyses. The ML phylogenetic tree was used as the working topology in CodeML analyses.

In this study, branch models were used to evaluate positive selection within the infraorder Brachyura. First, the one-ratio model (M0) estimated the distribution of the ω values as a baseline under the assumption that there is no adaptive evolution of the gene sequences, which assumes a single ω ratio for all branches of the phylogeny [25]. Then, positive selection acting on branches of interest was detected using a two-ratio model (M2), which allows the background and foreground lineages to have different ω ratio values [26,27]. Finally, the free-ratio model (M1), which allows the ω ratio variations between branches, was used to estimate the ω value on each branch [27]. The one-ratio model (M0) and the free-ratio model (M1) were compared to determine whether different clades in Brachyura had different ω values. The one-ratio model (M0) and the two-ratio model (M2) were compared to determine whether the ω values of the foreground and background branches were significantly different. In the one-ratio model, ω represents the value for the overall evolutionary tree. In the two-ratio model, ω_0_ and ω_1_ represent the values for the background branch and foreground branch, respectively. The paired models were compared with critical values of the Chi square (χ^2^) distribution using likelihood ratio tests (LRTs), where the test statistic was estimated to be twice the difference in log likelihood (2ΔL) and the degrees of freedom were estimated as the difference in the parameter number for each model.

In addition, we fit branch site models to check positive selection of some sites in the infraorder Brachyura. The branch-site models allow ω to vary between sites in proteins and between branches in trees. Branch-site model A (positive selection model) was used to identify the positively selected sites in Brachyuran species lineages (marked as foreground lineages). Comparing the significance level detection between Model A and the null Model with ω_2_ = 1 can be directly used to determine positive selection sites in the foreground Branch. The presence of sites with ω > 1 indicates that the data fitting of model A is significantly better than the corresponding null model. Bayes Empirical Bayes analysis was used to calculate a posterior probabilities in order to identify positive selection sites on the foreground lineages in the case of significant LRTs [28].

## 3. Results and Discussion

### 3.1. E. crenata Mitogenome Organization

**Figure 1:**
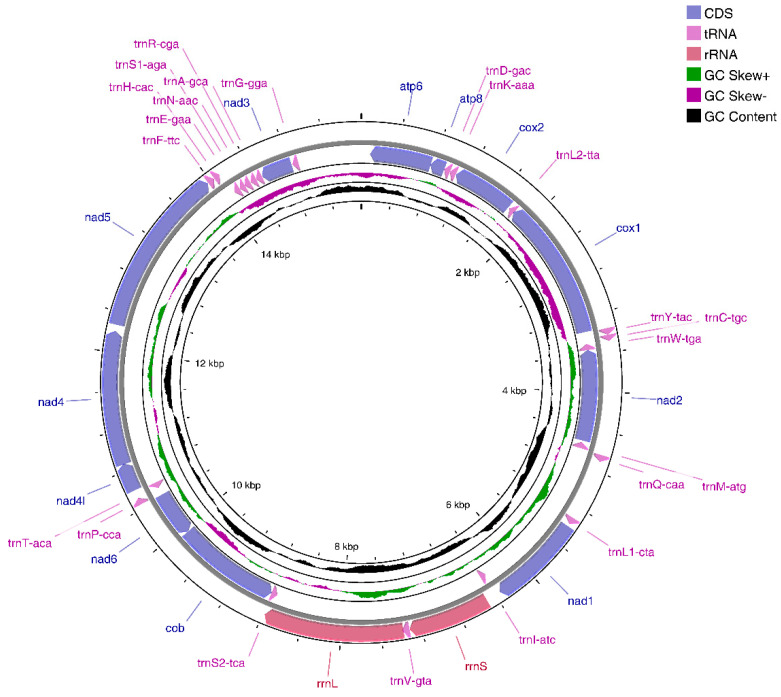
Complete mitogenome map of *E. crenata*. 14 genes are encoded on the heavy (H) strand and 23 genes are encoded on the light (L) strand. Genes for proteins and rRNAs are represented with standard abbreviations. Genes for tRNAs are shown in a single letter for the corresponding amino acid, with two leucine tRNAs and two serine tRNAs distinguished by numerals.

The Illumina NovaSeq runs resulted in 38,947,830 paired-end reads from the *E. crenata* library with an insert size of 350 bp. According to selective-assembly analysis, 167,844 reads were assembled into a 15,597 bp circular molecule (ON150678), which represented the complete mitogenome of *E. crenata* (Figure 1 and Table 2). This length is shorter than that of the complete mitogenome of most Brachyura crabs, including *Pseudohelice subquadrata*, *Macrophthalmus pacificus*, *Hemigrapsus penicillatus*, and *Grapsus tenuicrustatus* [29,30,31,32]. The genome encodes 37 genes, including 13 PCGs, two rRNA genes, and 22 tRNA genes (duplication of *trnL* and *trnS*). Metazoan mitogenomes usually have a control region (D-loop area), which varies in length between species. However, the D-loop area was not obvious in *E. crenata* mitogenome. As opposed to other Brachyura crabs [15,17], only 14 genes are found on the heavy (H) strand, whereas the other 23 genes are found on the light (L) strand. In addition, there are nine overlaps between adjacent genes in the *E. crenata* mitogenome with a length range of 1 to 46 bp (*cox3*/*atp6*, *atp6*/*atp8*, *trnL2-ttA*/*cox1*, *trnC-tgc*/*trnW-tga*, *trnW-tga*/*nad2*, *trnV-gta*/*rrnL*, *rrnL*/*trnS2-tca*, *cob*/*nad6*, *nad4l*/*nad4*) (Table 2). The *E. crenata* mitogenome also contains 941 bp intergenic spacers between genes in 20 regions ranging from 1 to 614 bp (Table 2), indicating the occurrence of tandem repeats and the loss of redundant genes.

Mitochondrial gene rearrangement is known as an effective phylogenetic signal for studying mitochondrial evolution [8]. Several studies and results have confirmed that gene rearrangements in metazoan mitochondrial genomes are conserved [33] and gene rearrangements occur relatively randomly and rarely [10,33,34,35]. However, it can be used as direct evidence of evolutionary relationships between species [36]. Gene order and strand arrangement in *E. crenata* is unidentical to that reported before in co-order species with mitochondrial genomes deposited in Genbank, i.e., *Perisesarma bidens*, *Parasesarma tripectinis*, and *Metopaulias depressus* [16,37,38]. After comparison and analysis with the ancestor of Decapoda [15], we found that *E. crenata* had a *trnH-cac* translocation, where the *trnH-cac* shifted into *trnE-gaa* and *trnF-ttc*, instead of the usual location between *nad5* and *nad4*. This phenomenon also occurs in several other species in Crustacea, such as *Grapsus albolineatus*, *Metopograpsus frontalis*, and *Macrophthalmus pacificus* [39,40,41]. As known, the tandem duplication/random loss model can explain the movement of *trnH-cac*, which is caused by tandem duplication in the region between *trnE-gaa* and *nad4*, followed by deletions of redundant genes that produce *trnH*-*trnF*-*nad5*.

The *E. crenata* mitogenome has a nucleotide composition of 36.19% A, 10.84% C, 16.90% G, and 36.07% T, with an overall AT content of 72.26% and GC content of 27.74%. This value of AT content was lower than the minimum value of 74.22% reported for *Parasesarma tripectinis* in the family Sesarmidae [36]. AT and GC skews are a measure of compositional asymmetry. The values of AT and GC skews found in *E. crenata* mitogenome were different in those of most animal mitogenomes. For *E. crenata*, the AT skew is 0.00166, and the GC skew is 0.218. As a representative of the family Goneplacidae, the AT skew and GC skew of *E. crenata* mitogenome showed different results with the species in the family Grapsidae, in which the AT skew and GC skew are always negative [15]. AT-skewed mitochondrial genomes are often reported in metazoan clades, such as crustaceans and brachyuran crabs [42,43,44], whereas GC-skewed mitochondrial genomes are not. The higher value of GC skew implies an apparent bias towards G in nucleotide composition.

### 3.2. Protein-Coding Genes

The PCGs include seven NADH dehydrogenases (*nad1*–*nad6* and *nad4l*), three cytochrome *c* oxidases (*cox1*–*cox3*), two ATPases (*atp6* and *atp8*), and one cytochrome *b* (*cob*). The total length of all 13 PCGs found of *E. crenata* is 11,049 bp, accounting for 70.8% of the complete length of mitogenome, and the PCGs encode 3683 amino acids (Table 2). Nine PCGs (*atp6*, *atp8*, *cob*, *cox1*, *cox2*, *cox3*, *nad2*, *nad3*, and *nad6*) are encoded on the light strand, while the remaining four (*nad1*, *nad4*, *nad4l*, and *nad5*) are encoded on the heavy strand. This transcriptional polarity is identical to the reported Brachyura mitochondrial genomes. Furthermore, there are two reading-frame overlaps on the same strand: *atp6* and *atp8*, and *nad4* and *nad4l* each share four and seven nucleotides (Table 2). Start and stop codons varied among the PCGs. Five of thirteen PCGs use ATG (*atp8*, *cox1*, *cob*, *nad4l* and *nad4*) and ATA (*atp6*, *cox2*, *nad2*, *nad1* and *nad5*), respectively, as a start codon, whereas *nad6* and *nad3* use ATT as a start codon. Nine PCGs use TAA (*atp6*, *cox2*, *cox1*, *nad1*, *nad6*, *nad4l*, *nad4*, *nad5* and *nad3*) as a stop codon and two PCGs use TAG (*atp8* and *nad2*). Finally, *cob* exhibit an incomplete (T) stop codon. An incomplete stop codon in the *cob* gene was also observed in the co-infraorder species *Chiromantes neglectum*, *Parasesarma affine*, *Parasesarma pictum*, and *Episesarma lafondii* [32,45,46,47]. It is assumed that truncated stop codons are completed via post-transcriptional polyadenylation [48]. Previous studies have shown that truncated stop codons were common in the metazoan mitogenomes and may be corrected by post-transcriptional polyadenylation [49,50].

**Figure 2:**
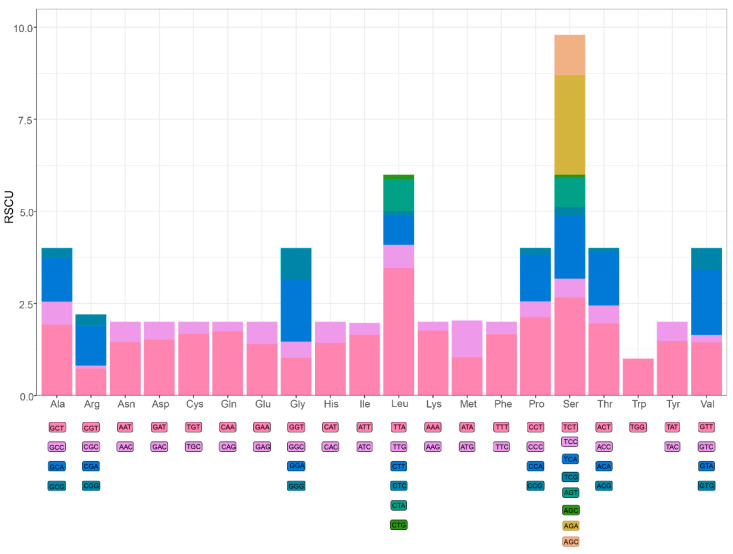
Relative synonymous codon usage (RSCU) of *E. crenata* mitochondrial PCGs. Numbers to the left mean the RSCU values. Codon families are represented on the X axis.

A large number of studies have shown that metazoan mitogenomes always have a bias towards higher representation of nucleotides A and T, resulting in subsequent bias in the corresponding encoded amino acids [51,52,53,54]. In the mitogenome of *E. crenata*, the A+T contents of PCGs and third codon positions are 69.87% and 69.70%, respectively. The amino acid usage and relative synonymous codon usage (RSCU) values in the PCGs of *E. crenata* are summarized in Figure 2. The mitogenome encodes a total of 3683 amino acids, among which serine (16.1%) and tryptophan (1.6%) are the most and the least frequently used, respectively. The RSCU values indicate that the six most commonly used codons are TTA (Leu), AGA (Ser), TCT (Ser), CCT (Pro), ACT (Thr), GCT (Ala) (Figure 2). Based on RSCU and amino acid composition in the PCGs, comparative analyses showed that the codon usage pattern of *E. crenata* is not conserved, varying from those of most Brachyuran species, such as *Grapsus albolineatus* and *Metopaulias depressus* [15,16]. In particular, leucine (9.8%) was found to be used less than serine, and AGA (Ser) plays a key role in the codon usage.

### 3.3. Ribosomal RNA and Transfer RNA Genes

The *rrnL* and *rrnS* genes of *E. crenata* are 1398 bp and 819 bp in length, respectively. Consistent with many Brachyura crabs [15,16], *rrnL* and *rrnS* are typically separated by *trnV* (*gta*) (Table 2). The AT and GC content of rRNAs are 78.3% and 21.7%, and the AT skew and GC skew are 0.022 and 0.29, respectively, suggesting an apparent bias toward the use of A and G.

Twenty-two tRNA genes were identified in the mitogenome of *E. crenata*, which is typical for metazoans. As opposed to most Brachyura crabs, i.e., *Eurypanopeus depressus*, *Panopeus herbstii*, *Rhithropanopeus harrisii* and *Grapsus albolineatus* [15,17], only eight of them are encoded by the heavy strand, and the rest are encoded on the light strand. The total length of tRNAs was 1477 bp, accounting for 9.47% of the mitogenome, and the length of these genes ranges from 62 (*trnC-tgc*) to 73 (*trnV-gta*) bp (Table 2). The regions contained 3.06 times more A–T content (75.36%) than G–C content (24.64%). Both AT skew and GC skew were positive, and their values were 0.035 and 0.11, respectively, showing a slight bias toward the use of A and an apparent bias toward G. In addition, the codons with A and T in the third position are used more frequently than other synonymous codons. These characteristics reflect codon usage with A and T bias at the third codon position, similar to the bias found in many metazoans [55,56,57].

### 3.4. Phylogenetic Relationships

**Figure 3:**
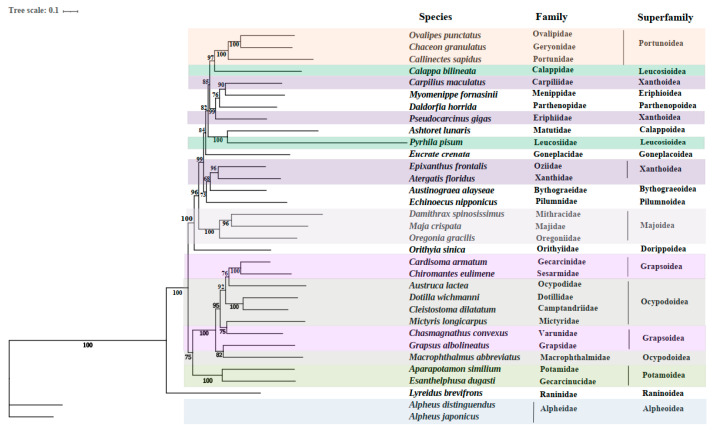
Phylogenetic tree derived from Maximum likelihood (ML) analysis based on nucleotide sequences of 13 mitochondrial PCGs (*atp6*, *atp8*, *cox1*, *cox2*, *cox3*, *cob*, *nad1*, *nad2*, *nad3*, *nad4*, *nad4l*, *nad5* and *nad6*). Species belonging to the same superfamily are marked with the same color. Numbers on branches represent ML bootstrap values.

In the present study, the phylogenetic relationships were analyzed based on the sequences of the 13 PCGs to clarify the relationships in *E. crenata* and 33 other known Brachyuran species (Figure 3). Phylogenetic analyses were carried out based on nucleotide sequences of 13 mitochondrial PCGs (*atp6*, *atp8*, *cox1*, *cox2*, *cox3*, *cob*, *nad1*, *nad2*, *nad3*, *nad4*, *nad4l*, *nad5*, and *nad6*) by the ML method. In the phylogenetic tree, some species from the same superfamily such as Xanthoidea, Grapsoidea, and Leucosioidea are not closest related, which may be caused by the selection methods of species here or differences in gene expression and morphological classification. As for the family Goneplacidae, it united well with Matutidae and Leucosiidae, and this also confirms a phylogenetic position of the family Goneplacidae close to Matutidae and Leucosiidae in the infraorder Brachyura. Among the 33 species included in the phylogenetic tree, each species in the tree has a high nodal support value in the clade. It is obvious that *Ashtoret lunaris* in the family Matutidae is closely related to *E. crenata*. *Phyrhila pisum* belonging to the family Leucosiidae also reveals a close relationship with *E. crenata*, which is second only to *Ashtoret lunaris*. This may improve the understanding of the phylogenetic position of *E. crenata* in Brachyura.

As shown in the previous study [58], *Ashtoret lunaris* also possesses a *trnH-cac* translocation. That is, the *trnH-cac* shifted into *trnE-gaa* and *trnF-ttc*. *Cob* exhibits an incomplete (T) stop codon, as does *E. crenata*. According to data analysis based on the *Ashtoret lunaris* genome sequence, serine (13.8%) and tryptophan (0.9%) are the most and the least frequently used, respectively. The similar rearrangements and codon usage between *Ashtoret lunaris* and *E. crenata* support the results above and provide further evidence for their close phylogenetic relationship.

### 3.5. Positive Selection Analysis

Purifying selection is the dominant force in mitotic evolution. Weak and/or episodic positive selection may occur under the strong purifying selection of reduced oxygen availability or greater energy requirements, because mitochondria are the primary site of aerobic respiration and are essential for energy metabolism [59,60,61]. Numerous studies have demonstrated that mitochondrial PCGs experienced positive selection in animals that survived in low oxygen environments, such as Tibetan humans, Ordovician bivalves, diving cetaceans, and insects [62,63,64,65].

Potential positive selection in *E. crenata* using CodeML implemented in the PAML package was examined to investigate whether marine ecosystems influence the function of mitochondrial genes. In the analysis of branch models, the ω (dN/dS) ratio that calculated under the one-ratio model (M0) was 0.03928 for the 13 mitochondrial PCGs of *E. crenata* (Table 3), indicating that these genes have underwent constrained selection pressure to maintain the main function. In the two-ratio model, ω_0_ and ω_1_, which represent the values for the background branch and foreground branch, were 0.04084 and 999.00000, respectively. The ω ratio of the foreground branch (ω_1_ = 999.00000) was significantly more than 1, indicating a strong positive selection of *E. crenata* by the infraorder Brachyura. In addition, many studies have shown that positive selection usually occurs over a short evolutionary time and performed on only a few sites [59,66]. Therefore, the signals of positive selection are often overwhelmed by those for successive purifying selection that occur at most sites in the gene sequence [59,61,66]. In the current study, branch-site models were used to determine possible positively selected sites in *E. crenata* (Table 4). One residue, located in *cox1*, was identified as a positively selected site with high BEB value (>95%). It is known that mitochondrial PCGs are crucial in the oxidative phosphorylation pathway. Cytochrome *c* oxidase catalyzes the oxygen terminal reduction, and the catalytic core is encoded by three mitochondrial PCGs (*cox1*, *cox2* and *cox3*) [61,67]. Cytochrome *c* oxidase has been confirmed to be a particularly important site of the positive selection in marine anoxic adaptation [68]. It has been reported that the cytochrome *c* oxidase requires reactive oxygen species (ROS) when the living cells are exposed to anoxia, and the increase in ROS concentration contributes to stabilize Hif-1α, which then results in the induction of Hif-1-dependent nuclear hypoxic genes [61,69,70]. The positive selection site in *cox1* suggests a phylogenetic adaption of ATP synthase in the presence of reduced oxygen availability or increased energy requirements. In addition, *cox1* in mitochondrion is involved in the regulation of platelet aggregation and vasomotor to maintain the stability of physiological functions of cells, tissues, and organs [61]. For *E. crenata*, functional modification mediated by positive selection mutations may increase the affinity between the enzyme and oxygen, and then efficiently utilize oxygen utilization under hypoxia conditions and maintain basic metabolic levels.

It’s well known that the marine environment is features with a lack of high pressure, variable temperatures, and low dissolved oxygen. Several studies have confirmed that all the above environmental factors could influence the mitochondrial aerobic respiration process [56,71,72]. In this study, the potentially adaptive residue was identified in the *cox1* gene, supporting the adaptive evolution of the *E. crenata* mitogenome. Our results provide an important basis for understanding how marine crabs maintain aerobic respiration to obtain adequate energy in marine chemosynthesis environment at the mitochondrial level.

## 4. Conclusions

In this study, the mitogenome of *E. crenata* was sequenced by HTS sequencing, thereby the first complete mitogenome for the family Goneplacidae was revealed. The *E. crenata* mitogenome is a typical closed-circular animal molecule including 13 PCGs, 22 tRNA genes, and two rRNA genes. Different from the published results for most Brachyura crabs, the AT skew and GC skew in the mitogenome are both positive. *E. crenata* shows a *trnH-cac* translocation between *trnE-gaa* and *trnF-ttc*. The phylogenetic analyses indicate that *E*. *crenata* is closely related to *Ashtoret lunaris* of Matutidae. Positive selection analysis showed that one residue located in *cox1* was identified as a positively selected site with high BEB value, which may suggest that this gene was under positive selection pressure in the marine ecosystem. The findings of this study could help to deepen the understanding of the phylogeny of Goneplacidae and the adaptive evolution at the mitochondrial level for marine crabs.

## Figures and Tables

**Table 1:** List of Brachyuran species with their GenBank accession numbers.

Superfamily	Family	Species	Size (bp)	Accession. no
Bythograeoidea	Bythograeidae	*Austinograea alayseae*	15,620	NC_020314
Calappoidea	Matutidae	*Ashtoret lunaris*	15,807	NC_024435
Dorippoidea	Orithyiidae	*Orithyia sinica*	15,568	NC_039639
Eriphioidea	Menippidae	*Myomenippe fornasinii*	15,658	NC_024437
Goneplacoidea	Goneplacidae	*Eucrate crenata*	15,597	ON150678
Leucosioidea	Calappidae	*Calappa bilineata*	15,606	NC_047195
	Leucosiidae	*Pyrhila pisum*	15,516	NC_030047
Majoidea	Majidae	*Maja crispate*	16,592	NC_035424
	Mithracidae	*Damithrax spinosissimus*	15,817	NC_025518
	Oregoniidae	*Oregonia gracilis*	15,737	NC_057204
Parthenopoidea	Parthenopidae	*Daldorfia horrida*	15,737	NC_049029
Pilumnoidea	Pilumnidae	*Echinoecus nipponicus*	16,173	NC_039618
Portunoidea	Geryonidae	*Chaceon granulatus*	16,135	NC_023476
	Ovalipidae	*Ovalipes punctatus*	16,084	NC_042695
	Portunidae	*Callinectes sapidus*	16,263	NC_006281
Potamoidea	Gecarcinucidae	*Esanthelphusa dugasti*	19,437	NC_060554
	Potamidae	*Aparapotamon similium*	19,236	NC_053821
Xanthoidea	Carpiliidae	*Carpilius maculatus*	15,761	NC_049030
	Eriphiidae	*Pseudocarcinus gigas*	15,515	NC_006891
	Oziidae	*Epixanthus frontalis*	15,993	NC_039110
	Xanthidae	*Atergatis floridus*	16,180	NC_037201
Raninoidea	Raninidae	*Lyreidus brevifrons*	16,112	NC_026721
Grapsoidea	Gecarcinidae	*Cardisoma armatum*	15,586	NC_057477
	Grapsidae	*Grapsus albolineatus*	15,578	NC_057301
	Sesarmidae	*Chiromantes eulimene*	15,894	NC_047209
	Varunidae	*Chasmagnathus convexus*	15,107	NC_052834
Ocypodoidea	Camptandriidae	*Cleistostoma dilatatum*	15,444	NC_060620
	Dotillidae	*Dotilla wichmanni*	15,600	NC_038180
	Macrophthalmidae	*Macrophthalmus abbreviatus*	16,322	NC_057472
	Mictyridae	*Mictyris longicarpus*	15,548	NC_025325
	Ocypodidae	*Austruca lactea*	15,659	NC_042401
Outgroup	Alpheidae	*Alpheus distinguendus*	15,700	NC_014883
		*Alpheus japonicus*	16,619	NC_038116

**Table 2:** Mitogenome organization of *E. crenata*.

Gene	Strand	Position	Length (bp)	Intergenic_spacer	Start_coden	Stop_coden	Anticodon
*cox3*	R	1–96	96	-	-	-	
*atp6*	R	96–767	672	−1	ATA	TAA	
*atp8*	R	764–922	159	−4	ATG	TAG	
*trnD-gac*	R	923–990	68	0	-	-	GAC
*trnK-aaa*	R	991–1059	69	0	-	-	AAA
*cox2*	R	1064–1735	672	4	ATA	TAA	
*trnL2-tta*	R	1754–1818	65	18	-	-	UUA
*cox1*	R	1814–3352	1539	−5	ATG	TAA	
*trnY-tac*	F	3362–3427	66	9	-	-	UAC
*trnC-tgc*	F	3428–3489	62	0	-	-	UGC
*trnW-tga*	R	3482–3549	68	−8	-	-	UGA
*nad2*	R	3548–4537	990	−2	ATA	TAG	
*trnM-atg*	R	4556–4623	68	18	-	-	AUG
*trnQ-caa*	F	4630–4698	69	6	-	-	CAA
*trnL1-cta*	F	5313–5380	68	614	-	-	CUA
*nad1*	F	5409–6347	939	28	ATA	TAA	
*trnI-atc*	R	6371–6436	66	23	-	-	AUC
*rrnS*	F	6500–7318	819	63	-	-	
*trnV-gta*	F	7320–7392	73	1	-	-	GUA
*rrnL*	F	7381–8778	1398	−12	-	-	
*trnS2-tca*	R	8733–8800	68	−46	-	-	UCA
*cob*	R	>8801–9935	1135	0	ATG	T	
*nad6*	R	9935–10,426	492	−1	ATT	TAA	
*trnP-cca*	F	10,444–10,510	67	17	-	-	CCA
*trnT-aca*	R	10,511–10,574	64	0	-	-	ACA
*nad4l*	F	10,595–10,879	285	20	ATG	TAA	
*nad4*	F	10,873–12,207	1335	−7	ATG	TAA	
*nad5*	F	12,271–13,965	1695	63	ATA	TAA	
*trnF-ttc*	F	13,972–14,039	68	6	-	-	UUC
*trnH-cac*	F	14,046–14,109	64	6	-	-	CAC
*trnE-gaa*	R	14,136–14,203	68	26	-	-	GAA
*trnS1-aga*	R	14,204–14,269	66	0	-	-	AGA
*trnN-aac*	R	14,270–14,337	68	0	-	-	AAC
*trnR-cga*	R	14,344–14,409	66	6	-	-	CGA
*trnA-gca*	R	14,411–14,478	68	1	-	-	GCA
*nad3*	R	14,482–14,826	345	3	ATT	TAA	
*trnG-gga*	R	14,836–14,903	68	9	-	-	GGA

**Table 3:** CodeML analyses of selection pressure on mitochondrial genes in *Eucrate crenata*.

Trees	Models	lnL	Parameter Estimates	Model Compared	2ΔL	LRT *p*-Value
Branch models						
ML tree	M0	−211,767.521149	ω = 0.04109			
	Two-ratio	−211,744.724139	ω0 = 0.04084 ω1 = 999.00000	Two-ratio vs. M0	45.5940200000186	0
	Free-tatio	−210,762.85387		Free-ratio vs. M0	2009.33455800003	0
Branch-sits models						
ML tree	Null model	−207,044.061032	p0 = 0.76754; p1 = 0.09376; p2a = 0.12361; p2b = 0.01510			
			ω0 = 0.03453; ω1 = 1.00000; ω2a = 1.00000; ω2b = 1.00000			
	Model A	−207,042.434633	p0 = 0.87845; p1 = 0.10727; p2a = 0.01272; p2b = 0.00155	Model A vs. null model	3.25279800000134	0.071301646
			ω0 = 0.03452; ω1 = 1.00000; ω2a = 20.67481; ω2b = 20.67481			

**Table 4:** Possible sites under positive selection in *Eucrate crenata*.

ML Tree			
Gene	Codon	Amino Acid	BEB Values
*atp6*	607	L	0.884
*cox1*	1389	V	0.984 *
*cox1*	1459	S	0.888
*cox1*	1469	T	0.887
*cox1*	1828	V	0.876
*cox1*	1961	L	0.829
*cox1*	2015	A	0.876
*cox1*	2021	T	0.512
*cox1*	2033	S	0.826
*cox2*	2344	M	0.86
*cox2*	2411	S	0.769
*cox2*	2611	A	0.863
*cox2*	2955	S	0.908
*cox3*	3274	L	0.632
*cox3*	3308	F	0.886
*cox3*	3469	V	0.569
*cox3*	3475	L	0.535
*cox3*	3511	L	0.534
*cox3*	3515	S	0.841
*cox3*	3605	S	0.53

* 0.95 < BEB < 0.99.

## Data Availability

The mitochondrial genome has been deposited in the NCBI with accession number ON150678.

## References

[1] Martijn J., Vosseberg J., Guy L., Offre P., Ettema T.J.G. (2018). Deep mitochondrial origin outside the sampled alphaproteobacteria. Nature.

[2] Boore J.L., Brown W.M. (1998). Big trees from little genomes: Mitochondrial gene order as a phylogenetic tool. Curr. Opin. Genet. Dev..

[3] Boore J.L., Macey J.R., Medina M. (2005). Sequencing and comparing whole mitochondrial genomes of animals. Method Enzymol..

[4] Boore J.L., Lavrov D.V., Brown W.M. (1998). Gene translocation links insects and crustaceans. Nature.

[5] Boore J.L. (1999). Animal mitochondrial genomes. Nucleic Acids Res..

[6] Kundu S., Kumar V., Tyagi K., Chakraborty R., Chandra K. (2019). The first complete mitochondrial genome of the Indian tent turtle, *Pangshura tentoria* (Testudines: Geoemydidae): Characterization and comparative analysis. Ecol. Evol..

[7] Dhar D., Dey D., Basu S., Fortunato H. (2020). Understanding the adaptive evolution of mitochondrial genomes in intertidal chitons. BioRxiv.

[8] Tan M.H., Gan H.M., Lee Y.P., Linton S., Grandjean F., Bartholomei-Santos M.L., Miller A.D., Austin C.M. (2018). ORDER within the chaos: Insights into phylogenetic relationships within the Anomura (Crustacea: Decapoda) from mitochondrial sequences and gene order rearrangements. Mol. Phylogenet. Evol..

[9] Schuster A., Vargas S., Knapp I.S., Pomponi S.A., Toonen R.J., Erpenbeck D., Worheide G. (2018). Divergence times in demosponges (Porifera): First insights from new mitogenomes and the inclusion of fossils in a birthdeath clock model. BMC Evol. Biol..

[10] Basso A., Babbucci M., Pauletto M., Riginella E., Patarnello T., Negrisolo E. (2017). The highly rearranged mitochondrial genomes of the crabs *Maja crispata* and *Maja squinado* (Majidae) and gene order evolution in Brachyura. Sci. Rep..

[11] Ji Y.K., Wang A., Lu X.L., Song D.H., Jin Y.H., Lu J.J., Sun H.Y. (2014). Mitochondrial genomes of two Brachyuran crabs (Crustacea: Decapoda) and phylogenetic analysis. J. Crustacean Biol..

[12] Castro P. (2007). A reappraisal of the family Goneplacidae MacLeay, 1838 (Crustacea, Decapoda, Brachyura) and revision of the subfamily Goneplacinae, with the description of 10 new genera and 18 new species. Zoosystema.

[13] Karasawa H., Kato H. (2001). The systematic status of the genus Miosesarma Karasawa, 1989 with a phylogenetic analysis within the family Grapsidae and a review of fossil records (Crustacea: Decapoda: Brachyura). Paleontol. Res..

[14] Ng P.K.L., Castro P. (2020). A revision of Carcinoplax abyssicola (Miers, 1885) and seven related species of Carcinoplax, H. Milne Edwards, 1852, with the description of two new species and an updated key to the genus (Crustacea, Decapoda, Brachyura, Goneplacidae). Zoosystema.

[15] Lu J.Y., Xia L.P., Liu X.J., Ma Y.W., Li J.J., Ye Y.Y., Guo B.Y. (2022). The mitochondrial genome of *Grapsus albolineatus* (Decapoda: Brachyura: Grapsidae) and phylogenetic associations in Brachyura. Sci. Rep..

[16] Rodriguez-Pilco M.A., Lesny P., Podsiadlowski L., Schubart C.D., Baeza J.A. (2022). Characterization of the Complete Mitochondrial Genome of the Bromeliad Crab *Metopaulias depressus* (Rathbun, 1896) (Crustacea: Decapoda: Brachyura: Sesarmidae). Genes.

[17] Jennings L.A., Blakeslee A.M.H., McCoy K.A., Behringer D.C., Bojko J. (2021). Systematic assessment of the Panopeidae and broader Eubrachyura (Decapoda: Brachyura) using mitochondrial genomics. Arthropod Syst. Phylo..

[18] Nayak A., Roy M.K.D., Mohanty B., Rout S.S., Dash B., Raman A.V., Patnaik L., Raut D. (2020). On the occurrence of an Euryplacid crab *Eucrate crenata* (De Haan, 1835), a first record from Odisha, India. Indian J. Geo-Mar. Sci..

[19] Lim H.J., Lee E.H., Yoon Y., Chua B., Son A. (2016). Portable lysis apparatus for rapid single-step DNA extraction of *Bacillus subtilis*. J. Appl. Microbiol..

[20] Bolger A.M., Lohse M., Usadel B. (2014). Trimmomatic: A flexible trimmer for Illumina sequence data. Bioinformatics.

[21] Perna N.T., Kocher T.D. (1995). Patterns of nucleotide composition at fourfold degenerate sites of animal mitochondrial genomes. J. Mol. Evol..

[22] Ohta T. (1992). The nearly neutral theory of molecular evolution. Annu. Rev. Ecol. S..

[23] Yang Z.H. (1998). Likelihood ratio tests for detecting positive selection and application to primate lysozyme evolution. Mol. Biol. Evol..

[24] Yang Z.H. (2007). PAML 4, a program package for phylogenetic analysis by maximum likelihood. Mol. Biol. Evol..

[25] Yang Z.H., Nielsen R., Goldman N., Pedersen A.M.K. (2000). Codon-substitution models for heterogeneous selection pressure at amino acid sites. Genetics.

[26] Nielsen R., Yang Z.H. (1998). Likelihood models for detecting positively selected amino acid sites and applications to the HIV-1 envelope gene. Genetics.

[27] Yang Z.H., Wong W.S.W., Nielsen R. (2005). Bayes empirical bayes inference of amino acid sites under positive selection. Mol. Biol. Evol..

[28] Xu X.D., Wu X.Y., Yu Z.N. (2010). The mitogenome of *Paphia euglypta* (Bivalvia: Veneridae) and comparative mitogenomic analyses of three venerids. Genome.

[29] Kim H.S., Kim K.Y., Lee S.H., Hong S.S., Cho I.Y., Yi C.H., Kim I.H., Yoon M., Kim M.S. (2019). The complete mitochondrial genome of *Pseudohelice subquadrata* (Dana, 1851) (Crustacea: Decapoda: Varunidae). Mitochondrial DNA B.

[30] Wang Q., Tang D., Guo H.Y., Wang J., Xu X.Y., Wang Z.F. (2020). Comparative mitochondrial genomic analysis of *Macrophthalmus pacificus* and insights into the phylogeny of the Ocypodoidea & Grapsoidea. Genomics.

[31] Karagozlu M.Z., Kim J.I., Choi T.J., Dinh T.D., Kim C.B. (2018). The complete mitochondrial genome of *Hemigrapsus penicillatus* (De Haan, 1835) (Decapoda, Varunidae). Mitochondrial DNA B.

[32] Sung J.M., Lee J., Kim S.K., Karagozlu M.Z., Kim C.B. (2016). The complete mitochondrial genome of *Grapsus tenuicrustatus* (Herbst, 1783) (Decapoda, Grapsidae). Mitochondrial DNA B.

[33] Wang Z.F., Wang Z.Q., Shi X.J., Wu Q., Tao Y.T., Guo H.Y., Ji C.Y., Bai Y.Z. (2018). Complete mitochondrial genome of *Parasesarma affine* (Brachyura: Sesarmidae): Gene rearrangements in Sesarmidae and phylogenetic analysis of the Brachyura. Int. J. Biol. Macromol..

[34] Zhang Y., Gong L., Lu X.T., Jiang L.H., Liu B.J., Liu L.Q., Lu Z.M., Li P.F., Zhang X. (2020). Gene rearrangements in the mitochondrial genome of *Chiromantes eulimene* (Brachyura: Sesarmidae) and phylogenetic implications for Brachyura. Int. J. Biol. Macromol..

[35] Zhang K., Zhu K.H., Liu Y.F., Zhang H., Gong L., Jiang L.H., Liu L.Q., Lu Z.M., Liu B.J. (2021). Novel gene rearrangement in the mitochondrial genome of *Muraenesox cinereus* and the phylogenetic relationship of Anguilliformes. Sci. Rep..

[36] Gong L., Lu X.T., Wang Z.F., Zhu K.H., Liu L.Q., Jiang L.H., Lu Z.M., Liu B.J. (2020). Novel gene rearrangement in the mitochondrial genome of *Coenobita brevimanus* (Anomura: Coenobitidae) and phylogenetic implications for Anomura. Genomics.

[37] Park Y.J., Park C.E., Lee S.H., Ko H.S., Ullah I., Hwang U.W., Shin J.H. (2018). The complete mitochondrial genome sequence of the intertidal crab *Parasesarma tripectinis* (Arthropoda, Decapoda, Sesarmidae). Mitochondrial DNA B.

[38] Li Y.T., Xin Z.Z., Tang Y.Y., Yang T.T., Tang B.P., Sun Y., Zhang D.Z., Zhou C.L., Liu Q.N., Yu X.M. (2020). Comparative mitochondrial genome analyses of sesarmid and other brachyuran crabs reveal gene rearrangements and phylogeny. Front. Genet..

[39] Kennish R., Williams G.A. (1997). Feeding preferences of the herbivorous crab *Grapsus albolineatus*: The differential influence of algal nutrient content and morphology. Mar. Ecol. Prog. Ser..

[40] Guan M.Y., Liu X.M., Lin F., Xie Z.F., Fazhan H., Ikhwanuddin M., Tan H.Q., Ma H.Y. (2018). The whole mitochondrial genome of the mangrove crab, *Metopograpsus frontalis* (Miers, 1880) (Decapoda, Grapsidae) and its phylogenetic relationship. Mitochondrial DNA B.

[41] Wang Z.Q., Shi X.J., Guo H.Y., Tang D., Bai Y.Z., Wang Z.F. (2020). Characterization of the complete mitochondrial genome of Uca lacteus and comparison with other Brachyuran crabs. Genomics.

[42] Gan H.Y., Gan H.M., Tan M.H., Lee Y.P., Austin C.M. (2016). The complete mitogenome of the hermit crab *Clibanarius infraspinatus* (Hilgendorf, 1869) (*Crustacea*: *Decapoda*: *Diogenidae*)—A new gene order for the Decapoda. Mitochondrial DNA A.

[43] Tang B.P., Xin Z.Z., Liu Y., Zhang D.Z., Wang Z.F., Zhang H.B., Chai X.Y., Zhou C.L., Liu Q.N. (2017). The complete mitochondrial genome of *Sesarmops sinensis* reveals gene rearrangements and phylogenetic relationships in Brachyura. PLoS ONE.

[44] Yang J.S., Nagasawa H., Fujiwara Y., Tsuchida S., Yang W.J. (2010). The complete mitogenome of the hydrothermal vent crab *Gandalfus yunohana* (Crustacea: Decapoda: Brachyura): A link between the Bythograeoidea and Xanthoidea. Zool. Scr..

[45] Xing Y.H., Ma X.P., Wei Y.Q., Pan D., Liu W.L., Sun H.Y. (2016). The complete mitochondrial genome of the semiterrestrial crab, *Chiromantes neglectum* (Eubrachyura: Grapsoidea: Sesarmidae). Mitochondrial DNA B.

[46] Wang Z.F., Shi X.J., Tao Y.T., Wu Q., Bai Y.Z., Guo H.Y., Tang D. (2019). The complete mitochondrial genome of *Parasesarma pictum* (Brachyura: Grapsoidea: Sesarmidae) and comparison with other Brachyuran crabs. Genomics.

[47] Zhang Y., Gao Y., Gong L., Lu X.T., Jiang L.H., Liu B.J., Liu L.Q., Lu Z.M., Li P.F. (2021). Mitochondrial Genome of *Episesarma lafondii* (Brachyura: Sesarmidae) and Comparison with Other Sesarmid Crabs. J. Ocean. U. China.

[48] Baeza J.A. (2018). The complete mitochondrial genome of the Caribbean spiny lobster *Panulirus argus*. Sci. Rep..

[49] Ojala D., Montoya J., Attardi G. (1981). TRNA punctuation model of RNA processing in human mitochondria. Nature.

[50] Dreyer H., Steiner G. (2006). The complete sequences and gene organisation of the mitochondrial genome of the heterodont bivalves *Acanthocardia tuberculata* and *Hiatella arctica*–and the first record for a putative Atpase subunit 8 gene in marine bivalves. Front. Zool..

[51] Salvato P., Simonato M., Battisti A., Negrisolo E. (2008). The complete mitochondrial genome of the bag-shelter moth *Ochrogaster lunifer* (Lepidoptera, Notodontidae). BMC Genom..

[52] Yu H., Li Q. (2012). Complete mitochondrial DNA sequence of *Crassostrea nippona*: Comparative and phylogenomic studies on seven commercial Crassostrea species. Mol. Biol. Rep..

[53] Wang Z.L., Li C., Fang W.Y., Yu X.P. (2016). The complete mitochondrial genome of two *Tetragnatha Spiders* (Araneae: Tetragnathidae): Severe truncation of tRNAs and novel gene rearrangements in araneae. Int. J. Biol. Sci..

[54] Sun S.E., Sha Z.L., Wang Y.R. (2018). Complete mitochondrial genome of the first deep-sea spongicolid shrimp *Spongiocaris panglao* (Decapoda: Stenopodidea): Novel gene arrangement and the phylogenetic position and origin of Stenopodidea. Gene.

[55] Chai H.N., Du Y.Z., Zhai B.P. (2012). Characterization of the complete mitochondrial genome of *Cnaphalocrocis medinalis* and *Chilo suppressalis* (Lepidoptera: Pyralidae). Int. J. Biol. Sci..

[56] Zhang B., Zhang Y.H., Wang X., Zhang H.X., Lin Q. (2017). The mitochondrial genome of a sea anemone *Bolocera sp.* exhibits novel genetic structures potentially involved in adaptation to the deep-sea environment. Ecol. Evol..

[57] Sun S.E., Hui M., Wang M.X., Sha Z.L. (2018). The complete mitochondrial genome of the alvinocaridid shrimp *Shinkaicaris leurokolos* (Decapoda, Caridea): Insight into the mitochondrial genetic basis of deep-sea hydrothermal vent adaptation in the shrimp. Comp. Biochem. Phys. D.

[58] Tan M.H., Gan H.M., Lee Y.P., Austin C.M. (2016). The complete mitogenome of the moon crab *Ashtoret lunaris* (Forskal, 1775), (Crustacea; Decapoda; Matutidae). Mitochondrial DNA Part A.

[59] Shen Y.Y., Liang L., Zhu Z.H., Zhou W.P., Irwin D.M., Zhang Y.P. (2010). Adaptive evolution of energy metabolism genes and the origin of flight in bats. Proc. Natl. Acad. Sci. USA.

[60] Tomasco I.H., Lessa E.P. (2011). The evolution of mitochondrial genomes in subterranean caviomorph rodents: Adaptation against a background of purifying selection. Mol. Phylogenet. Evol..

[61] Yang M., Gong L., Sui J.X., Li X.Z. (2019). The complete mitochondrial genome of *Calyptogena marissinica* (Heterodonta: Veneroida: Vesicomyidae): Insight into the deep-sea adaptive evolution of vesicomyids. PLoS ONE.

[62] Da Fonseca R.R., Johnson W.E., O’Brien S.J., Ramos M.J., Antunes A. (2008). The adaptive evolution of the mammalian mitochondrial genome. BMC Genom..

[63] Gu M.L., Dong X.Q., Shi L., Shi L., Lin K.Q., Huang X.Q., Chu J.Y. (2012). Differences in mtDNA whole sequence between Tibetan and Han populations suggesting adaptive selection to high altitude. Gene.

[64] Yang Y.X., Xu S.X., Xu J.X., Guo Y., Yang G. (2014). Adaptive evolution of mitochondrial energy metabolism genes associated with increased energy demand in flying insects. PLoS ONE.

[65] Plazzi F., Puccio G., Passamonti M. (2017). Burrowers from the past: Mitochondrial signatures of Ordovician bivalve infaunalization. Genome Biol. Evol..

[66] Zhang J.Z., Nielsen R., Yang Z.H. (2005). Evaluation of an improved branch-site likelihood method for detecting positive selection at the molecular level. Mol. Biol. Evol..

[67] Luo Y.J., Gao W.X., Gao Y.Q., Tang S., Huang Q.Y., Tan X.L., Chen J., Huang T.S. (2008). Mitochondrial genome analysis of Ochotona curzoniae and implication of cytochrome *c* oxidase in hypoxic adaptation. Mitochondrion.

[68] Mahalingam S., McClelland G.B., Scott G.R. (2017). Evolved changes in the intracellular distribution and physiology of muscle mitochondria in high-altitude native deer mice. J. Physiol..

[69] Chandel N.S., Maltepe E., Goldwasser E., Mathieu C.E., Simon M.C., Schumacker P.T. (1998). Mitochondrial reactive oxygen species trigger hypoxia-induced transcription. Proc. Natl. Acad. Sci. USA.

[70] Dirmeier R., O’Brien K.M., Engle M., Dodd A., Spears E., Poyton R.O. (2002). Exposure of yeast cells to anoxia induces transient oxidative stress-Implications for the induction of hypoxic genes. J. Biol. Chem..

[71] Mu W.D., Liu J., Zhang H.B. (2018). Complete mitochondrial genome of *Benthodytes marianensis* (Holothuroidea: Elasipodida: Psychropotidae): Insight into deep sea adaptation in the sea cucumber. PLoS ONE.

[72] Mu W.D., Liu J., Zhang H.B. (2018). The first complete mitochondrial genome of the Mariana Trench *Freyastera benthophila* (Asteroidea: Brisingida: Brisingidae) allows insights into the deep-sea adaptive evolution of Brisingida. Ecol. Evol..

